# Unbalanced Immune System: Immunodeficiencies and Autoimmunity

**DOI:** 10.3389/fped.2016.00107

**Published:** 2016-10-06

**Authors:** Giuliana Giardino, Vera Gallo, Rosaria Prencipe, Giovanni Gaudino, Roberta Romano, Marco De Cataldis, Paola Lorello, Loredana Palamaro, Chiara Di Giacomo, Donatella Capalbo, Emilia Cirillo, Roberta D’Assante, Claudio Pignata

**Affiliations:** ^1^Department of Translational Medical Sciences, Federico II University of Naples, Naples, Italy

**Keywords:** autoimmunity, immunodeficiency, autoimmune hemolytic anemia, immune thrombocytopenia, systemic lupus erytematous

## Abstract

Increased risk of developing autoimmune manifestations has been identified in different primary immunodeficiencies (PIDs). In such conditions, autoimmunity and immune deficiency represent intertwined phenomena that reflect inadequate immune function. Autoimmunity in PIDs may be caused by different mechanisms, including defects of tolerance to self-antigens and persistent stimulation as a result of the inability to eradicate antigens. This general immune dysregulation leads to compensatory and exaggerated chronic inflammatory responses that lead to tissue damage and autoimmunity. Each PID may be characterized by distinct, peculiar autoimmune manifestations. Moreover, different pathogenetic mechanisms may underlie autoimmunity in PID. In this review, the main autoimmune manifestations observed in different PID, including humoral immunodeficiencies, combined immunodeficiencies, and syndromes with immunodeficiencies, are summarized. When possible, the pathogenetic mechanism underlying autoimmunity in a specific PID has been explained.

## Introduction

Immunodeficiencies and autoimmunity may be considered two opposite conditions, deriving from different alterations of the immune system. However, the evidence that primary immunodeficiencies (PIDs) are often associated with different autoimmune manifestations suggests that they could share common pathogenetic mechanisms, which result in a broad immune dysregulation.

Immune system becomes self-tolerant through two main mechanisms called central and peripheral tolerance. As for T cells, central tolerance takes place within the thymus and is mediated by medullary thymic epithelial cells (mTEC), which express tissue-specific antigens under the control of the the transcription factor autoimmune regulator (AIRE) (1–3). Developing T-cells recognizing self-antigens receive a signal to die via programed cell death and, thereby, are deleted, through negative selection, from the T-cell repertoire (4, 5). As for B cells, negative selection of autoreactive cells takes place within the bone-marrow. Different mechanisms, including immunological ignorance, anergy, and suppression through regulatory T cells (Treg) are implicated in the control of self-reactive cells, which escape central tolerance and reach the periphery. For example, the ligation of T-cell receptor (TCR), in the absence of costimulatory molecules, makes the cells inable to express effector functions like cytokine secretion, leading to anergy (6). The control of the expression of the costimulatory molecules CD80 and CD86 is a major mechanism of peripheral tolerance (6).

In some cases, the inability to eradicate foreign antigens may lead to an exaggerated chronic inflammatory responses and autoimmunity (7–10), through several mechanisms, including molecular mimicry, by-stander activation, epitope spreading, and cryptic antigens.

Each PID is characterized by distinct, peculiar autoimmune manifestations (Tables 1 and 2), but the mechanisms may differ.

**Table 1:** **Autoimmune manifestations in humoral immunodeficiencies**.

1. SIgAD
Juvenile idiopathic arthritis Rheumatoid arthritis ITP, AHA IBD Sjogren’s disease Polyarteritis nodosa SLE Celiac disease T1D
2. CVID
ITP, AHA SLE IBD
3. PRKCD deficiency
Glomerulonephritis Polychondritis Antiphospholipid syndrome LES
4. LRBA deficiency
IBD AHA, ITP Granulomatous-lymphocytic interstitial lung disease T1D Neutropenia Chronic autoimmune hepatitis Eczema Uveitis Alopecia
5. Hyper-IgM syndrome
IBD Seronegative arthritis Hypothyroidism SLE Autoimmune hepatitis ITP, AHA T1D Uveitis

*SIgAD, selective IgA deficiency; ITP, immune thrombocytopenia, AHA, autoimmune hemolytic anemia; IBD, inflammatory bowel disease; SLE, systemic lupus eritematous; T1D, type 1 diabetes mellitus; CVID, common variable immunodeficiency*.

**Table 2:** **Autoimmune manifestations in combined immunodeficiencies and in syndromes with immunodeficiency**.

1. RAG-1/2 deficiency RMRP, ADA, IL2RG, Artemis, DNA ligase IV, ZAP70, and IL7Ra deficiency
Omenn syndrome (erythrodermia, alopecia, hepatosplenomegaly, and lymphoadenopathy)
2. PNP-deficiency, and mutations of ADA, DNA ligase IV, Cernunnos, ORAI1 and STIM1, and hypomorphic RAG1 mutations
AHA, ITP
3. Wiskott–Aldrich
AHA Autoimmune neutropenia Vasculitis IgA nephropathy Polyarthritis IBD
4. DiGeorge syndrome
ITP, AHA Autoimmune arthritis Autoimmune hepatitis Vitiligo IDB Autoimmune endocrinopathy
5. Ataxia telangiectasia
Psoriasis Autoimmune thyroid disease
6. STAT1 gain of function
Autoimmune thyroid disease IPEX-like phenotype (eczema, enteropathy, T1D, hypothyroidism, and growth hormone insufficiency)
7. STAT3 gain of function
Early onset autoimmunity (neonatal diabetes, enteropathy, desquamative interstitial pneumonitis, and posterior uveitis)

*ITP, immune thrombocytopenia; AHA, autoimmune hemolytic anemia; IBD, inflammatory bowel disease*.

In this review, we will describe the main autoimmune manifestations observed in different PIDs, including humoral immunodeficiencies, combined immunodeficiencies, and syndromes with immunodeficiencies, and, when possible, we will try to explain the pathogenetic mechanism underlying autoimmunity in a specific PID.

## Autoimmunity in Humoral Immunodeficiencies

### Selective IgA Deficiency

Selective IgA deficiency (SIgAD) is the most common PID in humans (11). According to European Society for Immunodeficiencies (ESID) criteria, SIgAD is defined by the presence of serum IgA levels <0.07 g/l in the absence of IgG and IgM deficiencies, after the age of 4 years (12). Patients with SIgAD have an increased risk to develop allergies and autoimmune manifestations, including juvenile idiopathic arthritis, rheumatoid arthritis, thrombocytopenic purpura, hemolytic anemia, inflammatory bowel disease (IBD), Sjogren’s disease, polyarteritis nodosa, systemic lupus erythematosus (SLE), celiac disease, and insulin-dependent diabetes mellitus (T1D) (Table 1) (13). Little is known about the pathogenesis of SIgAD and the predisposition to autoimmunity in these patients. Specific human leukocyte antigen (HLA) haplotypes, including 8.1, DR7, DQ2, DR1, and DQ5 have been identified in patients with SIgAD at higher risk autoimmune diseases (14), such as SLE, autoimmune thyroiditis, and celiac disease. In a recent study, the identification of single nucleotide polymorphisms in the IF1H1 gene encoding for an interferon inducible RNA helicase 1 protein and of a mutation in the CLEC16A gene in SIgAD patients has been associated with the development of autoimmune manifestations (14). Jacob et al. hypothesized that IgA exerts a protective role against autoimmunity. In particular, the interaction between the Fc fragment of IgA receptor and the immunoreceptor tyrosine-based activation motif deactivates the pathways of immune response carrying this motif through a partial phosphorylation (15). Moreover, the evidence of antibodies to bovine milk proteins in over 60% of IgA deficient patients may help explaining the association between SIgAD and inflammatory diseases of gastrointestinal tract (16, 17).

### Common Variable Immunodeficiency

Common Variable immunodeficiency (CVID) is a heterogeneous group of disorders characterized by a primary antibody deficiency, usually manifesting between the second and fourth decades of life with a mean age at onset of 26.3 years (18). It is the second most common immunodeficiency with an estimated prevalence ranging from 0.073 to 0.977 living patients per 100,000 inhabitants (19). According to the ESID diagnostic criteria, CVID should be taken into account in presence of a marked decrease of IgG and IgA with or without low IgM levels (measured at least twice; <2SD of the normal levels for their age) (http://esid.org/Working-Parties/Registry/Diagnosis-criteria). Moreover, all of the following criteria should be fullfilled: poor antibody response to vaccines (and/or absent isohemagglutinins) or low switched memory B cells (<70% of age-related normal value); secondary causes of hypogammaglobulinaemia have been excluded diagnosis is established after the 4th year of life; no evidence of profound T-cell deficiency (http://esid.org/Working-Parties/Registry/Diagnosis-criteria). More than 25% of CVID patients develop autoimmune complications (18, 20). Other medical conditions may include gastrointestinal infectious or inflammatory disease, lymphadenopathy, splenomegaly, and hematological malignancies (21). Cytopenia is the most common manifestation. Immune thrombocytopenia (ITP) has been found in up to 14% of patients and autoimmune hemolytic anemia (AHA) in up to 7% (22). In most cases (about 60%), the cytopenia precedes the identification of hypogammaglobulinemia (23). SLE has been reported in some rare CVID patient (24), predominantly females (89%). In about 50% of patients, CVID developed within 5 years of the diagnosis of SLE (24). Some patients experience an improvement in SLE symptoms when hypogammaglobulinemia appears (24). Hypogammaglobulinemia can develop because of the use of immunosuppressive treatment (i.e., corticosteroids or immunosuppressants). Unlike CVID, the cessation of therapy should solve hypogammaglobulinaemia. Nevertheless, in some occasion, the duration of post-cessation hypogammaglobulinaemia can be very prolonged, making difficult to understand its origin (25). IBD has been reported in 6–10% of CVID patients (Table 1) (22). Many different alterations could help explain the predisposition to autoimmune manifestations. In a subgroup of CVID patients, IL-7 levels were found to be increased (26, 27). IL-7 plays a key role in the expansion of autoreactive T-cell clones in the lymphopenic host (26, 27). Moreover, reduced levels of switched memory B cells and increased levels of activated CD21-low B cells have been associated with autoimmune manifestations in CVID. Increased levels of CD21-low B cells have been identified in SLE, rheumatoid arthritis, and cryoglobulinemia, suggesting a role for these cells in the pathogenesis of autoimmunity (28–30). Most CVID patients present with elevated BAFF levels (27). Of note, increased BAFF levels sustain the expansion of CD21-low B cells in CVID (31). Moreover, studies show that overexpression of BAFF in mice leads to B-cell hyperplasia, splenomegaly, and autoimmunity (32, 33). Different genetic mutations, including TACI, ICOS, BAFF-R, CD20, and CD21 have been associated with increased risk of developing CVID (34–40). Among these genetic alterations, autoimmunity is most common in TACI alterations [18/50 (36%) vs. 112/490 (23%) in wt TACI CVID], in particular, heterozygous C104R mutations (11/20 patients, 55%) (41).

### PRKCD Deficiency

A CVID-like disorder associated with multiple features of immune dysregulation, including glomerulonephritis, lymphadenopathy, relapsing polychondritis, and antiphospholipid syndrome has been recently described in a 12-year-old patient born to consanguineous parents of Turkish origin (42). Genetic studies revealed a mutation of *PRKCD* gene, leading to a complete absence of the protein. PRKCD deficiency has also been reported in three siblings with LES (Table 1) (43). PRKCD plays a key role in the regulation of cell survival, proliferation, and apoptosis (44). PRKCD deficiency in mice seems to be related to a defective deletion of autoreactive B cells during B-cell development, due to impaired proapoptotic extracellular signal-regulated kinase signaling (45, 46).

### LRBA Deficiency

LPS-responsive beige-like anchor protein (LRBA) deficiency is a novel PID caused by either homozygous or compound heterozygous mutations in *LRBA* that abolish LRBA protein expression. This PID is characterized by early onset hypogammaglobulinemia, autoimmune manifestations, susceptibility to IBD, and recurrent infections (47). However, it has been also described in patients with IBD with or without antibody deficiency (48, 49), in patients with autoimmune manifestations without hypogammaglobulinemia (50), or in patients with immune dysregulation, polyendocrinopathy, enteropathy, and X-linked syndrome (IPEX)-like disorder (51). The main clinical manifestations of LRBA deficiency are immune dysregulation (95%), followed by organomegaly (86%) and recurrent infections (71%). The most common autoimmune manifestations are enteropathy (59%), AHA (50%), and ITP (50%). A lower number of patients presented granulomatous-lymphocytic interstitial lung disease (36%), T1D or neutropenia (22%), chronic autoimmune hepatitis (13%), eczema and uveitis (9%), and alopecia (4.5%) (Table 1). LRBA is a highly conserved multidomain protein implicated in regulating endosomal trafficking, cell proliferation, and survival. LRBA deficiency is associated with increased apoptosis and altered phenotype of Treg cells, which express lower levels of key effector proteins involved in Treg cell suppression, such as CD25 and CTLA-4. This results in decreased frequency, aberrant phenotype, and decreased suppressive function of such cells. These alterations might play a critical role in the ubiquitous autoimmune manifestations of the disease.

### CTLA-4 Haploinsufficiency

CTLA-4 haploinsufficiency has been recently associated with lymphoproliferation, lymphocytic infiltration, autoimmunity, peripheral B-cell lymphopenia, hypogammaglobulinemia, and increased CD21lo B cells (52). In mouse models, homozygous CTLA-4 deficiency leads to a lethal autoimmune phenotype characterized by multiorgan lymphocytic infiltration and destruction (53, 54) resambling FOXP3 deficiency (55–57). CTLA-4 plays a key role in immune tolerance. Recent studies show that CTLA-4 is able to suppress the expression of CD80 and CD86 from antigen presenting cells (APCs) via transendocytosis (58). The depletion of the costimulatory ligands reduces T cell activation (59).

### Activated Phosphoinositide 3-Kinase δ Syndrome

Activated phosphoinositide 3-kinase δ syndrome (APDS) 1 and 2 are PID resulting from autosomal dominant mutations in PI3KCD and PIK3R1, respectively (60, 61). Autoimmune manifestations are reported in 34% of APDS1 patients. The clinical manifestations included cytopenias (AHA or tri-lineage cytopenia), glomerulonephritis, exocrine pancreatic insufficiency, autoimmune thyroid disease, seronegative arthritis, recurrent pericarditis, sclerosing cholangitis, and gastrointestinal nodular mucosal lymphoid hyperplasia (61). Autoimmune manifestations have been reported in the 17% of the APDS2 patients. They included ITP, AHA, Evans syndrome, T1D, chronic arthritis, autoimmune hepatitis, and chronic eczema (60). PI3Kδ is implicated in the regulation of Treg cell function. Studies suggest that PI3K is an important target for the treatment of different autoimmune conditions.

### Hyper-IgM Syndrome

Hyper-IgM syndrome (HIGM) is a group of disorders characterized by alterations of immunoglobulin receptor isotype switching, leading to normal or elevated IgM antibody and very low IgA, IgG, and IgE antibodies (62). Alterations in different genes implicated in CD40-CD40L pathway involved in B cell activation, class switch recombination or somatic hypermutation have been identified in HIGM. Seven different forms of HIGM have been till now described. Most of the cases (65–70%) are due to mutations of the gene encoding for CD40 ligand (CD40L) on the X chromosome, leading to HIGM1 (63). The other forms are due to mutations of AID (HIGM2), CD40 (HIGM3), UNG (HIGM5), NEMO (HIGM6), and IkBα (HIGM7). No genetic defect has been so far identified for HIGM4.

Autoimmunity has been described in all forms of HIGM. HIGM1 patients have an increased risk to develop IBD, seronegative arthritis, hypothyroidism, and SLE (64). In the 21% of patients affected with HIGM2 autoimmune hepatitis, ITP, T1D, IDB, and uveitis have been described (65). In addition, in patients with NEMO defects, AHA, IBD, and arthritis have been described (Table 1) (66). Studies on transgenic mouse models suggest that CD40–CD40L interactions is involved in the elimination of autoreactive B cells (67). In fact, an increase of circulating polyreactive B cells and a significant decrease of CD25+Foxp3+Treg cells have been reported in CD40L-deficient patients suggesting defects of the peripheral B-cell tolerance mechanism. An imbalanced production of cytokines, including as IL-1, IL-8, IL-6, IL-10, IL-12, and tumor necrosis factor (TNF)-α may be observed in CD40-deficient patients (68). This impairment is the consequence of the involvement of CD40–CD40L interaction in T-cell dependent macrophage-mediated immune response, implicated in the maturation of dendritic cells and regulation of the T-cell activation. The transcription factor NFkB plays a key role in the regulation of pro-inflammatory responses. Recent studies suggest that gut epithelial cells are directly implicated in the control of epithelial integrity and the regulation of the interaction between the mucosal immune system and gut microflora. In mice, NEMO deficiency causes a severe chronic intestinal inflammation, which has been associated with apoptosis of colonic epithelial cells, impaired expression of antimicrobial peptides, and translocation of bacteria into the mucosa. The chronic inflammatory response observed within the colon, is dominated by innate immune cells, as suggested by the upregulation of IL1b, IL6, TNF, and Ccl2 and by the infiltration of large numbers of dendritic cells and granulocytes in the colon. Eventually, also T lymphocytes are involved, as suggested by the presence of lymphoid follicles and a massive infiltration with CD4+ T cells in the gut mucosa.

## Combined Immunodeficiecies

Severe combined immunodeficiency (SCID) is a group of different PIDs characterized by a severe deficiency of the cellular and humoral immune system. SCID phenotype may be due to a variety of different mutations. From a clinical point of view, SCID is characterized by recurrent severe infections, chronic diarrhea, and failure to thrive (69, 70). The clinical presentation may drive the diagnosis toward a specific molecular cause of SCID (69). Patients affected with SCID often develop autoimmune manifestations. This may appear surprising in that SCID patients, who are unable to mount any immune response to foreign pathogens, may paradoxically develop autoimmune phenomena. Alterations in both central and peripheral tolerance have been described in SCID patients (71).

### Autoimmunity in Omenn Syndrome

Omenn syndrome (OS) is a SCID inherited in an autosomal recessive manner, caused by homozygous or compound heterozygous mutations in recombinase activating gene 1 (RAG1) or RAG2, implicated in V(D)J recombination, which represents a crucial step in T- and B-cell differentiation. OS has also been associated with hypomorphic mutations in other different genes, including RMRP, ADA, IL2RG, Artemis, DNA ligase IV, ZAP70, and IL-7Ra deficiency (72, 73). Signs of OS, including oligoclonal T-cell expansion, generalized rash, and lymphadenopathy have been reported in some patient affected with DiGeorge syndrome. This rare condition is known as atypical complete DiGeorge syndrome (74). Apart from recurrent infections, patients affected with OS also show features of autoimmunity, including erythrodermia, alopecia, hepatosplenomegaly, and lymphadenopathy (Table 2). The hallmark of the syndrome is the expansion and activation of a peripheral oligoclonal population of autoreactive T cells, due to defective central (75, 76) and peripheral tolerance mechanisms (77). Studies suggest that in OS, defective AIRE expression may lead to inadequate expression of tissue-specific self-antigens by mTEC, impairing central tolerance. In these patients, the T-cell compartment is composed by a high proportion of autoreactive T cells, which are able to expand in peripheral tissues leading to the clinical symptoms. Similarly, alterations in central tolerance may be implicated in the pathogenesis of immune manifestations also in PIDs characterized by ineffective thymopoiesis, such as the DiGeorge syndrome or in SCID characterized by partial defects of the T-cell maturation, such as IL-7alfa, common γ chain, or ARTEMIS defects. The persistent infectious/inflammatory state and the presence of immunologic “space,” which increases the ability of T cells to respond to an excess of cytokines or antigens, impairs peripheral tolerance in SCID patients. Treg population may be also affected in SCID patients.

### Autoimmune Manifestations in SCID Due to IL7R Mutations

IL7Ra deficiency is responsible of the majority of T-B+NK+ cases (72) characterized by an increased susceptibility to severe and opportunistic infections. In a few cases, autoimmune manifestations have been reported (72, 73). Autoimmune manifestations presented with OS in one infant (73), and cytopenias in three other cases. Autoimmune cytopenias have been also described in some patients with PNP-deficiency, and mutations of ADA, DNA ligase IV, Cernunnos, and hypomorphic RAG1 mutations (Table 2) (69, 78–82).

### Ca++ Channellopathies Due to Mutations in ORAI1 and STIM1

Null or loss-of-function mutations in ORAI1 or STIM1 are associated with a SCID-like disease characterized by recurrent and chronic infections, autoimmunity, ectodermal dysplasia, and muscular hypotonia in the presence of numerically intact T, B, and NK cells. Symptoms usually manifest in the first year of life. Lymphoproliferation, AHA, and ITP are very common in patients with STIM1 mutations (Table 2). Autoimmunity may derive from alterations of negative selection of autoreactive T cells and/or B cells during their development. In fact, Ca2+ signals are implicated in TCR and BCR signaling and thus potentially influence the selection thresholds in immature T and B cells. Moreover, a reduced frequency of Treg cells has been observed in STIM1-deficient patients (83, 84) and in one patient with ORAI1 p.R91W mutation.

## Syndromes with Immunodeficiency

### Wiskott–Aldrich

Wiskott–Aldrich syndrome is a very rare immunodeficiency, characterized by thrombocytopenia, eczema, and recurrent bacterial infections appearing in the first months of life. Other features includes humoral and cellular immunodeficiency, defects of the innate immunity (85–87), increased risk to develop autoimmune manifestation and malignancies, impaired apoptosis (88, 89), and defective cell motility (90). The gene responsible for WAS (WASP) is located on the X chromosome and encode for WASP protein, which is only expressed in the cytoplasm of hematopoietic cells. WASP protein plays a major role in the transduction of the signals from the cell surface to the actin cytoskeleton, which regulates actin polymerizationand the formation of actin filament (91, 92). WAS patients are at a higher risk of developing autoimmunity and most of WAS patients (about 40%) are affected by at least one autoimmune manifestation (93, 94). The most common autoimmune manifestations include AHA, autoimmune neutropenia, vasculitis, and IgA nephropathy with or without the association with Henoch–Schönlein purpura, polyarthritis and IBD (Table 2) (87, 93–95). Studies suggest that a defect in Treg cells could be implicated in the pathogenesis of autoimmune manifestations (96, 97). In fact, Treg cells, isolated from WAS patients, show a reduced ability to suppress effector T-cell proliferation and IFN-γ production (98, 99). On the contrary, Treg cell development is not impaired in these patients. In addition, Treg cells from WASp−/− mouse show a reduced granzyme B secretion, which results in the inability to suppress B-cell proliferation and apoptosis. Furthermore, studies on mouse models show that Treg cells from WASp−/−mouse are not able to prevent the development of autoimmunity in scurfy mice (Foxp3-deficient) (98–100). Also B cells may be implicated in the pathogenesis of autoimmune manifestations in WAS patients. Studies show that selective deletion of WASP in B cells leads to the production of autoantibodies and the development of autoimmunity (101, 102).

### DiGeorge Syndrome

Autoimmune manifestations have been reported in about the 10% of patients with DiGeorge syndrome (103–105). Autoimmune disorders include mainly autoimmune cytopenias (ITP, AHA) (106–108), autoimmune arthritis (107), autoimmune hepatitis, vitiligo, IDB, and autoimmune endocrinopathy (Table 2) (109). Impaired T-cell development in an abnormal thymus may result in altered central tolerance and escape of self-reactive T. Thymic abnormality may also result in impaired generation of Treg (96, 110, 111).

### Ataxia Telangiectasia

Patients with ataxia telangiectasia (A-T) have increased frequency of autoimmune disorders (112), including psoriasis and autoimmune thyroid disease (Table 2). Loss of suppressor T-cell function has been described as responsible for the development of autoimmune disease.

### STAT1 Gain of Function

Increased incidence of autoimmunity has been reported in heterozygous STAT1 gain-of-function (GOF) mutations (113). The main clinical features of the syndrome include chronic mucocutaneous candidiasis (CMC) (114–118), disseminated coccidioidomycosis, and histoplasmosis (116, 119), recurrent sinopulmonary infections and pneumonias (with or without bronchiectasis), herpes virus infections, blood-borne infections, squamous cell cancer, and cerebral aneurysms (116, 120). The most common autoimmune manifestation is thyroiditis, but in some case patient may show an IPEX-like phenotype (121). Number and function of Treg cells are usually normal and the pathogenesis of IPEX-like disease remains unclear (121).

### STAT3 Gain of Function

Recent studies show that activating STAT3 mutations may lead to autoimmunity, hypogammaglobulinemia, lymphoproliferation, and mycobacterial disease (122). The autoimmune manifestations are early onset and include neonatal diabetes and some rare disorders, such as desquamative interstitial pneumonitis and posterior uveitis (123). Patients with activating STAT3 mutations show a reduced number of Th17 cells, decreased IL-17 production, and deficiency of Treg, NK, and dendritic cells (123). Autoimmunity may develop as a consequence of the impaired Treg development.

## Conclusion

Autoimmunity and immunodeficiencies represent two opposite conditions, which may coexist in the context of a general immune dysregulation. Even though different mechanisms have been identified to explain autoimmunity in PIDs, the pathogenesis of autoimmunity remains unexplained in most of the cases. Considering this strong association, underlying immunodifiency should be always excluded in particular in presence of early onset or multiple autoimmune manifestations (124).

## Author Contributions

All authors listed, have made substantial, direct and intellectual contribution to the work, and approved it for publication.

## Conflict of Interest Statement

The authors declare that the research was conducted in the absence of any commercial or financial relationships that could be construed as a potential conflict of interest.
